# Supplementary material for: Direct-written polymer field-effect transistors operating at 20 MHz
Source: Sci Rep. 2016 Dec 12;6:38941. doi: 10.1038/srep38941 (PMC5150525; doi:10.1038/srep38941)
Supplement: Supplementary Information [file srep38941-s1.pdf]

SUPPLEMENTARY INFORMATION

Direct-written polymer field-effect transistors operating at 20 MHz

Andrea Perinot[1,2], Prakash Kshirsagar[3], Maria Ada Malvindi[3], Pier Paolo Pompa[3,4], Roberto Fiammengo[3,*], Mario Caironi[1,*]

[1] Center for Nano Science and Technology@PoliMi, Istituto Italiano di Tecnologia, via Giovanni Pascoli 70/3, Milan, Italy

[2] Dipartimento di Fisica, Politecnico di Milano, Piazza Leonardo da Vinci 32, Milano, Italy

[3] Center for Biomolecular Nanotechnologies@UniLe, Istituto Italiano di Tecnologia (IIT),Via Barsanti, 73010 Arnesano, Lecce, Italy

[4] Istituto Italiano di Tecnologia, Via Morego 30, 16163 Genova, Italy

***Authors to whom correspondence should be addressed:** mario.caironi@iit.it, roberto.fiammengo@iit.it

SUPPLEMENTARY FIGURES

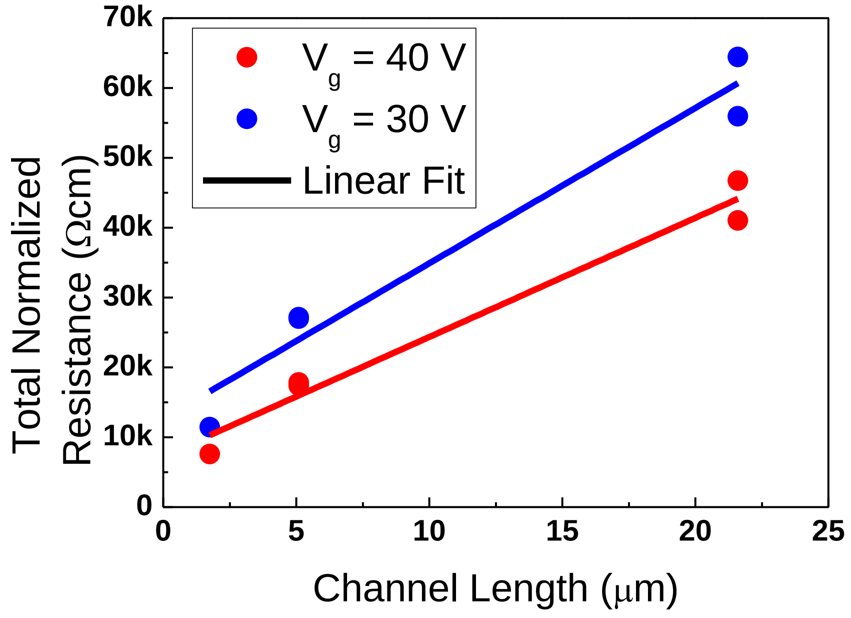

Figure S1. Determination of the contact resistance via TLM method

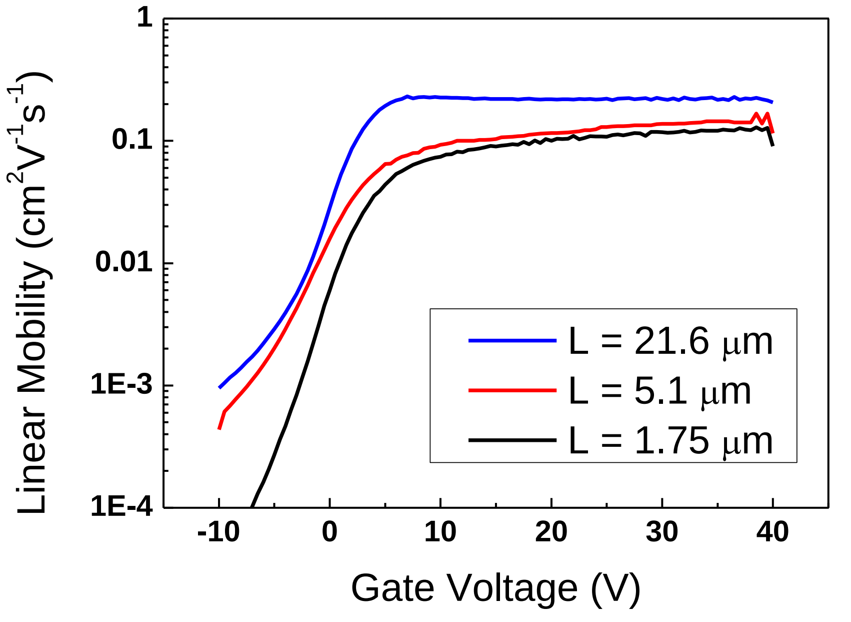

Figure S2. Apparent charge mobility extracted in the linear region for OFETs with different channel length

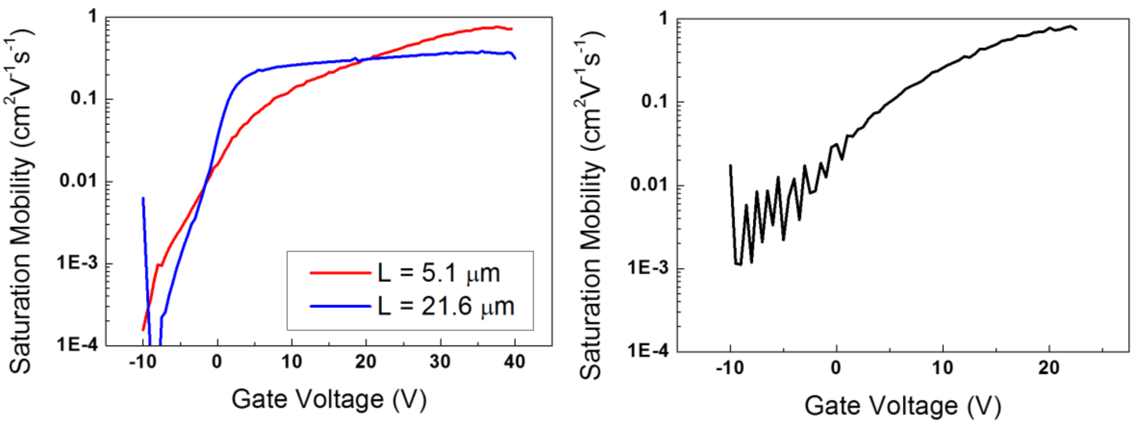

Figure S3. Apparent charge mobility extracted in the saturation region (V_d = 40 V) for OFETs with long (left) and short (L = 1.75 μm, right) channel length.

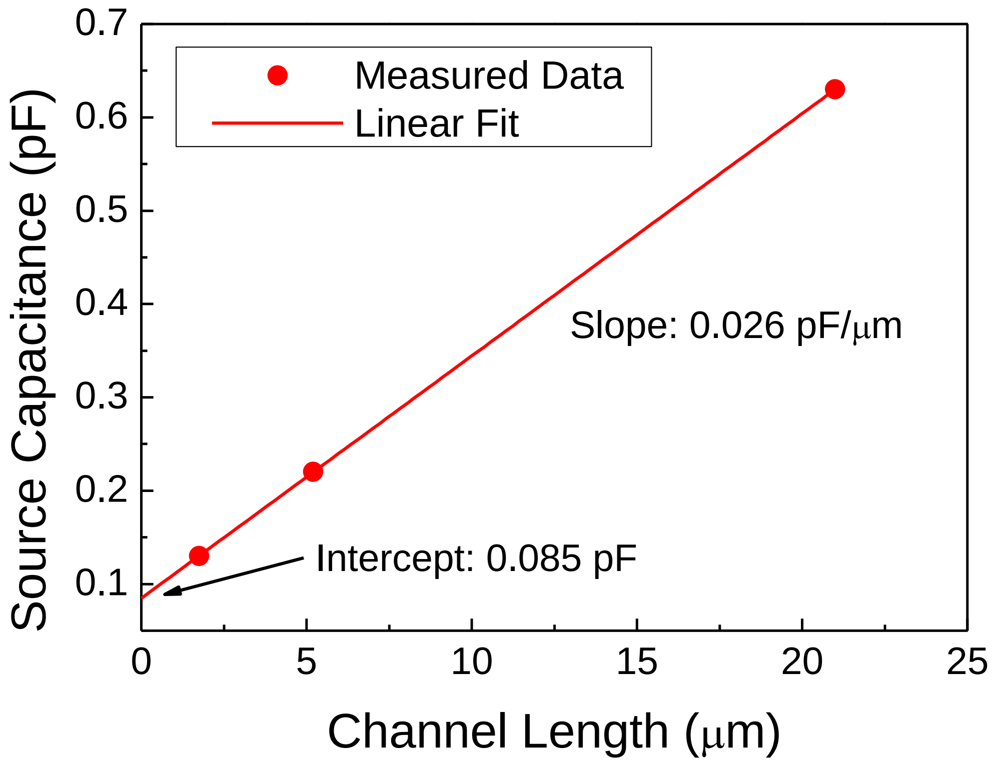

Figure S4. Measured gate-source capacitance in the saturation regime (V_g = V_d = 25 V) for different channel lengths and linear fit of the measured data.

SUPPLEMENTARY DISCUSSION

Theory of frequency operation of OFETs

Following the well-known gradual channel approximation model for Field-Effect Transistors, when modulating the gate-source voltage of a FET with a small signal v_{gs}, superimposed on a DC bias V_{GS}, we measure an AC current i_d at the drain terminal:

$$\boldsymbol{i_d = g_m\, v_{gs}}$$

The transconductance g_m is defined as follows, for the linear and saturation regimes of operation for the FET:

$$\boldsymbol{g_m = \frac{d\,(I_D + i_d)}{d\,(V_{GS} + v_{gs})} = \frac{\mu C_{diel} W}{L} V_{DS}}$$

$$\boldsymbol{g_m = \frac{d\,(I_D + i_d)}{d\,(V_{GS} + v_{gs})} = \frac{\mu C_{diel} W}{L} (V_{GS} + v_{gs} - V_T)}$$

The transconductance of an ideal FET is constant versus frequency, until the upper-bound limit, defined by the transit time of the charge carriers across the channel, is reached. However, in real devices, it is common that other parasitisms intervene before this upper bound is reached, and become the main determinants of the upper frequency limit for a FET. Among these, we consider here the capacive parasitisms introduced in the top-gate staggered configuration by the geometrical overlap between the gate contact and the source (C_{gs}) and drain (C_{gd}) contacts (Fig S5d), which comply with the following I-V relationship:

$$i = j2\pi f C v$$

A commonly used figure of merit for the characterization of the maximum operating frequency of a FET is the transition frequency f_t, which is defined by $\frac{i_d}{i_g} = 1$. By combination of this definition with the previous equations we derive:

$$f_t = \frac{g_m}{2\pi\,(C_{gs} + C_{gd})}$$

The transition frequency can also be extracted graphically, by plotting i_d and i_g on the same graph and identifying the crossing point between the two, which identifies $\frac{i_d}{i_g} = 1$.

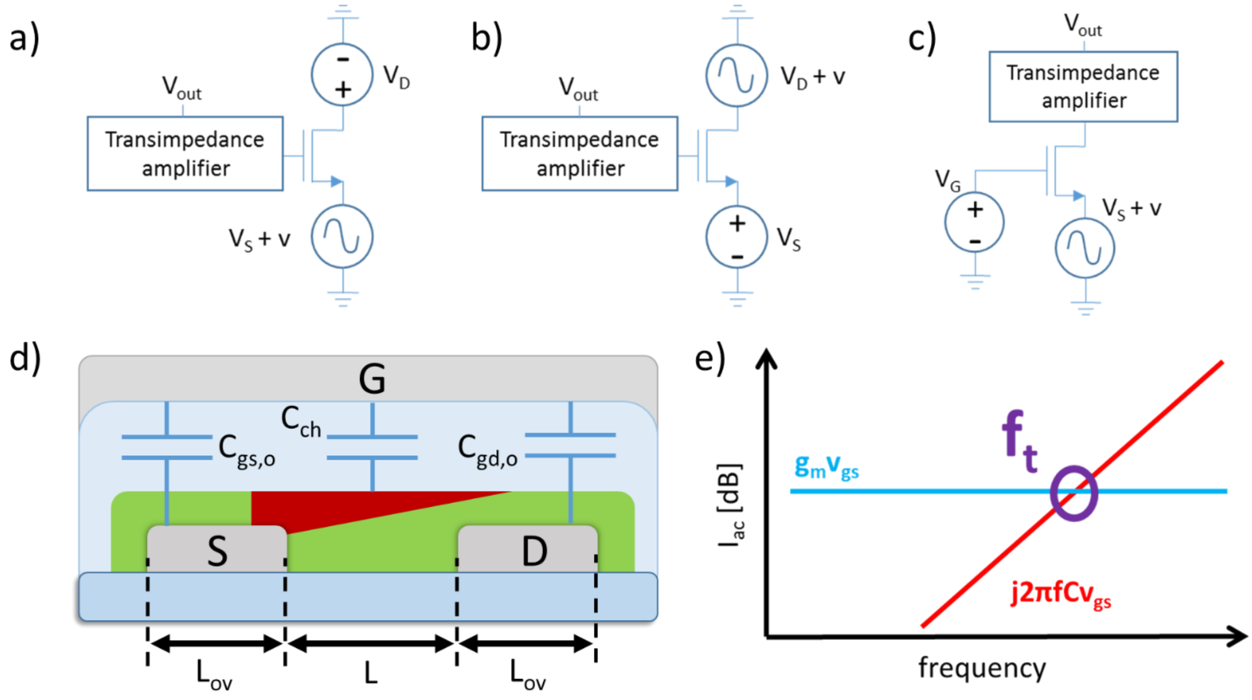

Figure S5. a),b),c) Measurement setup configurations for the measurement of Cgs, Cgd, and gm respectively, d) sketch of the lateral view of the realized OFETs, highlighting the different capacitances insisting across the device electrodes, e) sketch of the ideal behavior for the transconductance and capacitances measured for a typical OFET device, and determination of its transition frequency via the crossing-point method

Measurement setup

The FET parameters required for the calculation of the transition frequency were measured through a custom setup in which a Vector Network Analyzer (Agilent E5061B) feeds a sinusoidal signal superimposed to a DC bias into one of the transistor terminals, depending on the desired parameter to be measured. Then, after a transimpedance amplifier (FEMTO DHPCA100) collects the current flowing into another properly chosen FET terminal, the VNA reads the output signal and provides the desired transfer function. The three different

setup configurations used for the determination of $C_{gs,}$ C_{gd} and g_m are shown respectively in Fig S5a, S5b and S5c, the amplifier gain was set to 2 x 10^3, the sinusoidal signal was set to a fixed power of 0 dBm, and the maximum signal frequency was set to 2 MHz.

Ideal behavior

The configuration shown in Fig S5c enables the determination of the dynamic transconductance of the device. The RF signal v_{gs} is fed into the source terminal and the amplifier collects the current being driven into the drain terminal, and recalling the equations above it is straightforward that the transfer function between these two corresponds to g_m, after taking into account the gain introduced by the amplifier. To ensure a correct measurement of the current being fed into the actual device, it is necessary to notice that these configuration guarantees that no current is collected by the parasitic gate-drain capacitance, since the voltage drop across its terminals remains constant throughout the measurement.

The ideal transconductance for a FET, before charge carriers transit time limitations arise, is schematically illustrated by the blue line in Fig S5e.

The configurations shown in Fig S5a (Fig S5b) allow the determination of the capacitances insisting between the source (drain) and the gate terminals. In general, they include both a contribution from the geometrical overlap between the electrodes and a contribution from the charge accumulation in the channel region. The way these contributions are distributed between the measurements depends on the operation regime of the device: when operated in linear regime ($V_{\mathrm{ds}} \approx 0$ V) the channel capacitance is ideally equally split between the source and drain, while when operated in saturation ($V_{\mathrm{gs}} - V_{\mathrm{t}} = V_{\mathrm{ds}}$) the charge density is not uniform along the channel length direction (schematized in Fig S5d) and there is a low-resistance path only between the source terminal and the accumulated channel, so its capacitance is only measured when probing the source electrode. The ideal output for the capacitance measurements is schematized by the red line in Fig S5e, characterized by a slope of +20 dB/dec. Recalling from the previous discussion and referring to the same figure, if we plot

simultaneously g_m and $C_{gs} + C_{gd}$ the crossing point between the two curves identifies the actual transition frequency value for the device.
